# Colorectal cancer in Tanzania: the current status and future directions

**DOI:** 10.3332/ecancer.2023.1564

**Published:** 2023-06-22

**Authors:** Larry Akoko, Nathan Brand, Vihar Kotecha, Sam Byabato, Jerry Ndumbalo, Masumbuko Mwashambwa, Amos Mwakigonja, Ann Lowry

**Affiliations:** 1 Department of Surgery, Muhimbili University for Health and Allied Sciences, PO Box 65001, Dar es Salaam, Tanzania; 2 Department of Surgery, University of California San Francisco, San Francisco, CA 94115, USA; 3 Department of Surgery, Catholic University for Health and Allied Sciences, PO Box 1464, Mwanza, Tanzania; 4 Clinical Oncology Department, Ocean Road Cancer Institute, PO Box 3592, Dar es Salaam, Tanzania; 5 Department of Surgery, University of Dodoma School of Medicine and Dentistry, Dodoma 41218, Tanzania; 6 Department of Pathology, Muhimbili University for Health and Allied Sciences, PO Box 65001, Dar es Salaam, Tanzania; 7 Department of Surgery, University of Minnesota, Minneapolis, MN 55455, USA; † Larry Akoko and Nathan Brand contributed equally and are designated as co-first authors.

**Keywords:** colorectal cancer, global oncology, Tanzania, global surgery, global pathology, cancer in LMIC

## Abstract

**Introduction:**

Globally, colorectal cancer (CRC) is the third most common malignancy and the second most common cause of cancer death. By 2030, the incidence is expected to increase to reach 2.2 million cases and 1.1 million deaths. In Sub-Saharan Africa, accurate cancer incidence data is limited, but anecdotally, clinicians note a significant rise in the incidence of CRC in the past decade. To educate clinicians on the growing burden of CRC, the Tanzanian Surgical Association hosted a 4-day CRC symposium from 3rd to 6th October 2022. Following the meeting, a group of multidisciplinary stakeholders created a working group whose first task was to assess the epidemiology, presentation and available resources for CRC care in Tanzania. The findings of that assessment are described in this article.

**Findings:**

The true incidence of CRC in Tanzania is currently unknown. However, individual high-volume centres have noted a dramatic rise in cases of colon and rectal cancer on their wards. A review of the published data on CRC in Tanzania showed that most patients present with CRC late and the limited availability of endoscopic and diagnostic services poses a challenge for accurately staging these patients prior to treatment. Multidisciplinary care, including surgery, chemotherapy and radiation, is available for the treatment of CRC in Tanzania, although the capacity and quality of these services vary throughout the country.

**Conclusion:**

There is a substantial burden of CRC in Tanzania that appears to be increasing. While there is capacity in the country to provide all aspects of multidisciplinary care, late presentation, limited access to diagnostic and treatment services and poor coordination continue to be significant barriers to providing optimal treatment to these patients.

## Introduction

Globally, colorectal cancer (CRC) is the fourth most common malignancy and the third most common cause of cancer death [1]. By 2030 the incidence is estimated to reach 2.2 million cases and 1.1 million deaths [2, 3]. Currently, CRC is most common in areas with high human development indices (HDI) such as in Europe and North America. However, with improvement in life expectancy and HDI in many low- and middle-income countries, a rise in incidence is projected to occur.

In Sub-Saharan Africa specifically, accurate cancer incidence data is limited due to the lack of or low coverage of available population-based registries. Overall, high-quality population-based registries [4] cover only 2% of the population. Despite limited population-based data in Tanzania, clinicians anecdotally note a significant rise in the incidence of the disease. To stimulate clinicians' interest in CRC, the Tanzanian Surgical Association, with support from the International Cancer Institute, hosted a 4-day CRC symposium from 3–6 October 2022. The symposium aimed to update clinicians on the practice guidelines for the treatment of CRC and discuss opportunities to implement system-wide interventions to improve the care of patients with colon cancer in Tanzania. Forty-nine participants attended the event from 16 different institutions including all zonal referral hospitals in the country. In response to gaps identified during the symposium, a multidisciplinary working group was created to explore the opportunities to improve the multidisciplinary care of CRC in Tanzania. The first task of this group was to assess the epidemiology, presentation and available resources for CRC care in Tanzania through a review of the literature and publicly available data.

### Tanzania healthcare system

The most recent census in Tanzania shows that the population has now risen to 61.7 million people [5]. Geographically, the country is divided into 184 districts. These districts are organised into 31 regions which are in-turn organised into 8 zones throughout the country. The public healthcare system is organised in a similar manner with district, regional, zonal and national referral hospitals. In total, there is 1 national referral hospital, 5 zonal referral hospitals and 31 regional referral hospitals [6]. Payment for medical services is either out of pocket for uninsured patients or through private or national insurance. A vast majority of the insured patients utilise the national insurance scheme called the National Health Insurance Fund (NHIF); however, utilisation of this service is still low, with only 4,404,581 or 8% of the population having coverage from NHIF [7].

### Epidemiology of CRC in Tanzania

The true incidence of CRC in Africa, and specifically in Tanzania, is currently unknown. A recent meta-analysis of population-based cancer registries estimated that the age-standardised risk (ASR) of CRC in Africa is 5.25 per 100,000 persons [8]. However, none of the studies included in the meta-analysis were from Tanzania and all the studies from East Africa were published in 2000 or before. Because of these limitations, conclusions about the incidence of CRC in Tanzania should be interpreted with caution. However, individual cancer registries in the region report significant increases in rates of CRC. For example, a population-based registry from Harare, Zimbabwe described that the incidence of CRC has increased by 4% per year from 1991 to 2010 [4]. In Uganda, the Kampala cancer registry, a population-based registry that covers Kyadondo County where Kampala is located, found that from 1991 to 2015 the age-standardised rates of CRC increased from 6.8 to 11.0 per 100,000 in men and 4.9 to 9.2 per 100,000 women resulting in a 73% and 53% increase in men and woman respectively [9].

The two population-based cancer registries in Tanzania, the Mwanza Cancer Registry and the Kilimanjaro Cancer Registry (KCR) still lack the coverage to provide any conclusive data on trend. Established in 2016, The Mwanza cancer registry covers three districts surrounding the city of Mwanza: Nyamagana, Ilemela and Sengerema. Case finding in the very rural Sengerema district is still being optimised, but the ASR of CRC per 100,000 people among men and women from 2016 to 2017 was reported to be 3.2 and 2.6, respectively [10]. However, because this registry was very recently created, the authors suggested that these results should be viewed as ‘preliminary’[10]. The KCR was initially founded in 1974. Staffing shortages resulted in periods of dormancy; in 2018, the registry was placed under the management of the Kilimanjaro Cancer Center. They report the ASR for CRC within their catchment area to be 4.3 and 3.1 for men and woman respectively [10]. However, it should be noted, that the KCR only reported half of all the expected cancer cases based on extrapolated data from GLOBOCAN, suggesting that their surveillance may be incomplete.

While the two population-based cancer registries in Tanzania have not been reliably collecting data long enough to ascertain if there is a change in the incidence of colon cancer in Tanzania, several reports from the largest hospitals in the country suggest CRC is becoming more common on their wards. For example, Ocean Road Cancer Institute (ORCI) in Dar es Salaam, the largest cancer centre in Tanzania, examined the incidence of CRC from 2005 to 2015. They noted a rise in the frequency of CRC from 23 cases a year in 2005 to 146 cases per year in 2015 [11], representing a 6-fold increase for the decade. Similarly, a review of the cases from Kilimanjaro Christian Medical Center, in Northern Tanzania, showed that their CRC case volume increased from 15 to 20 a year in 1998 to close to 50 cases a year in 2018 [12]. While the available data does not conclusively determine what the ASR of CRC in Tanzania is, or if it is increasing, these reports from ORCI and KCMC suggest that for clinicians working at the national referral hospital and the zonal referral hospitals, are seeing more patients with CRC.

Late presentation is a common feature of CRC in Tanzania. In Bugando, a zonal referral hospital for the Lake and Western Zones in Tanzania, a review of 332 CRC cases from 2006 to 2011 reported that 100% of patients were symptomatic at presentation and their symptoms were evident for a median of 22 months. Furthermore, 13.5% of these cases presented as emergencies. Of the patients with CRC included in the study from Bugando, the stage at presentation was 3% Stage 1, 42% Stage 2, 30% Stage 3 and 25% Stage 4 [13]. Similarly, a review of 174 CRC patients from 1998 to 2018 at Kilimanjaro Christian Medical Center found that 65% of patients presented as an emergency, 28% had regional disease defined as involving lymph nodes or other structures and 34.3% had metastatic disease [12]. At ORCI, in a review of 122 patients from 2010 to 2015 identified no patient presenting with Stage 1 disease, 5% with Stage 2, 33% with Stage 3 and 62% with Stage 4; 100% of patients were symptomatic at the time of diagnosis [14].

In high-income settings, screening for CRC has demonstrated substantial benefit. In Europe, with well-developed national screening programs and participation rates of greater than 50%, screening detects CRC 40%–60% of cases [15]. Not surprisingly, screen detected cancer are significantly smaller proportion of CRC patients who present to the hospital in Tanzania. While lack of screening services might explain part of the late presentation, delays in the referral path and long distances to treatment facilities are possible additional factors.

### Diagnostics capacity

Availability of diagnostic services is vital to any oncology program to ensure accurate histological or cytological diagnosis, staging and selection of suitable treatment strategy. For CRC patients, endoscopic evaluation through flexible fibreoptic endoscopy or proctoscopy is the first step. Endoscopy ensures characterisation and biopsy of a suspicious lesion to allow an accurate diagnosis of CRC.

The next step is colonoscopy to identify synchronous cancers or adenomatous polyps. To our knowledge, there are no published reports on the availability of endoscopy services within mainland Tanzania. In Zanzibar, there is only a single colonoscopy unit at Mnazi Mmoja Hospital. A recent review of 448 patients who underwent colonoscopy at that unit from 2013 to 2021 found that CRC was identified in 18% of patients and colonic polyps in 25% [16]. At the CRC symposium, an informal survey of all attendees suggested that 75% of participants from 16 institutions throughout the country had colonoscopy services available at their institution. However, the lack of an on-site pathologist leads to prolonged turnaround time between colonoscopy and diagnosis of cancer. Importantly, it should be noted that the participants of the CRC symposium work primarily at tertiary facilities in major cities within the country so is not representative of the majority of rural hospitals in Tanzania.

Once tissue is obtained, pathology and imaging capacity are integral to the workup and treatment of patients with CRC. According to the Tanzania Cancer Treatment Guidelines, at minimum, patients with colon cancer need access to an X-ray for chest imaging, an abdominal ultrasound for peritoneal and liver assessment and pathology laboratory [17]. In addition, cross-sectional imaging, preferably with magnetic resonance imaging (MRI), is also required to accurately stage patients with rectal cancer. According to personal communications with the Tanzanian Ministry of Health, 43 computerized tomography (CT) scanners and 10 MRI machines are installed in public facilities in the country as response to COVID-19 outbreak. In comparison, Canada has 549 CT scanners and 378 MRI machines for a population of 38 million [18]; even with the new capacity, there is a clear need to scale up diagnostic radiology services for the 61.7 million people. Furthermore, in Tanzania, the development of rectal cancer MRI protocols is needed to better identify rectal cancer patients who might benefit from neoadjuvant chemoradiation.

**Figure 1.**
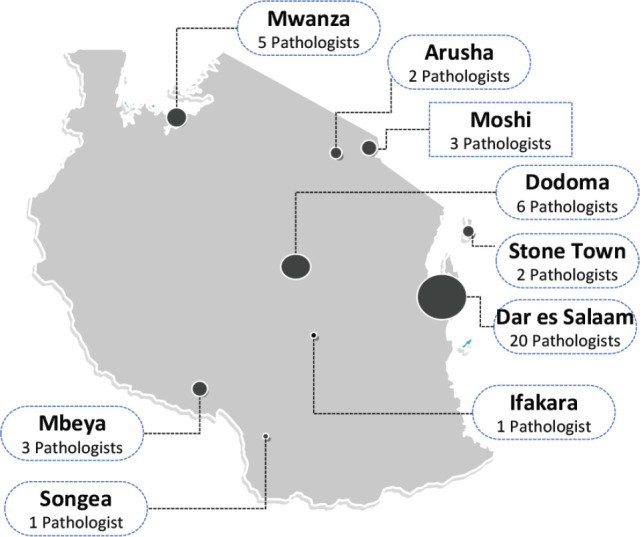
Number of working pathologists nationally in Tanzania. The number and location of working pathologists in both the public and private sector in Tanzania. Information obtained through private communications with the Tanzanian chapter of the Association of Pathologists of East Central and Southern Africa.

The availability of histopathology services in Tanzania has steadily increased since Tanzania gained independence in 1961. At that time, Tanzania had only one anatomical pathologist; now there are about 43 anatomical pathologists distributed in various hospitals and at least one stand-alone anatomical pathology lab. The distribution of pathologists and their labs is summarised in Figure 1.

Due to the limited access of diagnostic services in the country, it is unsurprising that a significant portion of patients with CRC in Tanzania receive treatment before being completely worked up for their disease. In the cohort of 174 patients from Kilimanjaro Christian Medical Center, it was found that only 39% of patients underwent a colonoscopy [12]. In Bugando, none of the 323 patients received a colonoscopy; however, 33% underwent proctosigmoidoscopy [13]. None of the cohorts reported the proportion of patients who received a carcinoembryonic antigen (CEA) level, so it is unclear how often this important tumour marker was utilised.

### Available treatments and outcomes

The treatment of CRC requires multidisciplinary care, including surgery, stoma care, chemotherapy and radiation depending on the location and stage of the disease. Other than stoma care, all these modalities are available in the public sector in Tanzania where most of the cancer patients are treated. Radiation services are available at two public facilities, ORCI in Dar es Salaam and Bugando Medical Center in Mwanza; and three private hospitals, two in Dar es Salaam, Agha Khan Hospital and Besta Hospital and Good Samaritan Cancer Hospital in Ifakara township in the Morogoro region. Two radiation units are under construction at an additional two public hospitals, Benjamin Mkapa Medical Center in Dodoma and KCMC in Moshi.

**Figure 2.**
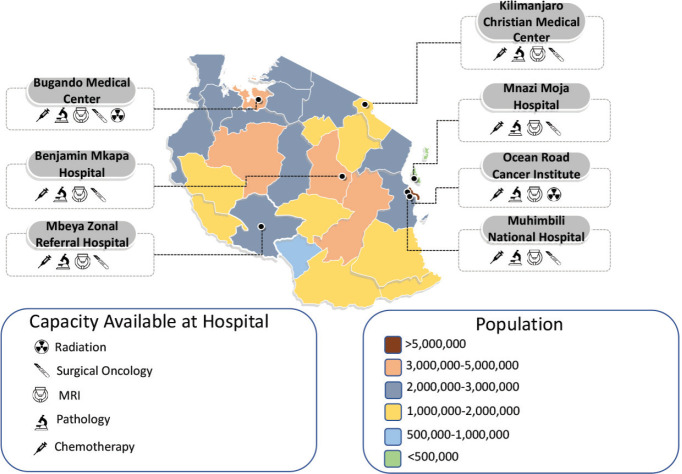
Public zonal and national referral hospital capacity and overall population in Tanzania. Capacity and location of the public hospitals in Tanzania overlaid on a map of the country with the current population per the 2022 Tanzanian census.

Chemotherapy is available at all the public Zonal referral hospitals in the major cities in Tanzania: Mbeya, Dodoma, Mwanza, Moshi, Dar es Salaam, Morogoro and Stone Town in Zanzibar. In addition, elective surgical resections are offered at all six of the zonal and National referral hospitals and a few private and faith-based institutions. These resections are all performed open by general surgeons, and the oncological adequacy of these resections regarding surgical margins and lymph node resections has yet to be published. The capacity of all public zonal and national referral hospitals is further described in Figure 2.

Finally, limited information on the medium and long-term outcomes of patients with CRC in Tanzania is available. Globocan data suggest that the mortality-to-incidence ratio of colon cancer in Tanzania is 0.67 compared to 0.35 in the United States [19]. Data from Bugando reported that of the 332 patients reviewed, 10.5% died during their initial hospitalisation. However, long-term survival could not be assessed as 85% of patients were lost to follow-up by 6 months post-discharge [13]. The lack of data highlights the need for further investment into prospective cohort studies that can identify the proportion of patients that are receiving a complete workup prior to treatment, guideline concordant care and adequate surgical management. In addition, these studies can provide long-term outcomes of patients with CRC in Tanzania, which is currently unknown.

### Challenges

At the CRC symposium, structured discussions on the challenges clinicians face when treating CRC patients in Tanzania highlighted several themes highlighted in Table 1. However, these gaps need to be better understood through systematic study to determine appropriate interventions to increase early detection.

The barriers identified by clinicians at the symposium mirror the findings of several quantitative studies of barriers and facilitators to cancer care in Tanzania. In 2019, in preparation for the rollout of the National Cancer Treatment Guidelines, Luhar *et al* [23] interviewed 21 oncology clinicians at ORCI. These interviews revealed a lack of capacity and infrastructure, and difficulty with multidisciplinary communication, including non-standardised operation notes, pathology reports and radiology reports. Similarly, mismanagement of cases was rampant, especially by practitioners from private facilities [20]. Structured interviews of 62 Tanzanian cancer patients identified similar themes and highlighted the significant delays in diagnosis faced by patients. The median interval between the first symptom and diagnosis for that cohort was 358 days. Significantly, 66% of these patients went directly to the healthcare system after their first symptom appeared, highlighting that significant delays occur after patients present to a healthcare facility. The remaining 44% delayed a median of 3 months [21].

The lack of public awareness of cancer as a disease is also a significant barrier in Tanzania. At Kilimanjaro Christian Medical Center, a survey of 245 newly diagnosed patients with cancer between 2018 and 2019 found that 71% of patients stated their failure to seek medical attention earlier was due to the lack of knowledge about cancer [22]. The same study reported that 66% of respondents stated that they ‘thought treatment might be too expensive’ as another explanation for the delay in seeking care, highlighting that fear of cost continues to be a significant barrier for patients [22].

Participants in the CRC symposium also identified a need for more clarity on standard approaches to the workup and treatment of CRC patients as a barrier to treatment in Tanzania. In 2020, the Tanzanian Ministry of Health published national cancer treatment guidelines for common cancers in Tanzania, including colon and rectal cancer [17]. These guidelines describe standard approaches to the staging workup and treatment of both colon and rectal cancer. However, there are gaps in the treatment guidelines including the absence of the standards of surgical resection for colon or rectal cancer and reporting standards for histology and radiology. In addition, awareness of the guidelines was limited among the symposium participants.

### The way forward

Based on the barriers identified, the decision was made to create a core multidisciplinary working group consisting of surgeons, radiologists, medical and radiation oncologists and pathologists. The priorities for the group are improving cancer registration in the country, the standardisation of treatment and improving interdisciplinary communication among different members of the healthcare system. To accomplish this, the group will meet regularly to establish guidelines for CRC surgery in Tanzania and create synoptic operative reports, pathology reports and radiology reports to minimise missing information necessary to make treatment decisions. In addition, the group hopes to develop mentorship programs for all multidisciplinary team members to support improving medical services outside the major cities where a large portion of the population is treated.

## Conclusion

There is currently a substantial burden of CRC in Tanzania that appears to be increasing over the past 10 years. While there is capacity in the country to provide all aspects of the multidisciplinary care required to treat CRC patients, late presentation, limited access to diagnostic and treatment services and poor coordination continue to be significant barriers to providing optimal treatment to these patients. Recently, the Tanzanian Surgical Association hosted a CRC symposium aimed to update clinicians on the practice guidelines for the treatment of CRC and discuss opportunities to implement system-wide interventions to improve the care of patients with colon cancer in Tanzania. Through this symposium, a multidisciplinary working group convened focused on improving the care of CRC patients within the country.

## Conflicts of interest

Nathan Brand was supported by the Fogarty International Center of the National Institutes of Health under Award Number D43TW009343 and the University of California Global Health Institute.

## Funding statement

This article was not funded by any organisation.

## Figures and Tables

**Table 1.** Barriers to evidence-based treatment of rectal cancer in Tanzania.

**Economic barriers**
High out-of-pocket costs for patients
**Patient barriers**
Lack of public awareness
Late-stage disease at diagnosis
**Healthcare barriers**
Low index of suspicion for cancer among primary care providers
Long wait times for endoscopy services
Long turn-around-time for pathologic results
**Organisational barriers**
Lack of functioning multidisciplinary management teams and tumour boards
Lack of clarity around standard treatments for rectal cancer
**Infrastructure barriers**
Lack of capacity to provide adequate staging of rectal cancer patients with MRI
Lack of specialised equipment to allow for safe oncologic rectal surgery
